# Preface 25 Years of Current Genomics

**DOI:** 10.2174/138920292501240205235837

**Published:** 2024-02-05

**Authors:** Fabio Coppedè

**Affiliations:** 1 Department of Translational Research and of New Surgical and Medical Technologies, University of Pisa, Pisa, Italy

   The journal Current Genomics is celebrating its twenty-fifth volume this year, and I am very honored to serve as Editor-in-Chief at this momentous time. The journal was launched in 2000 under the leadership of Prof. Stefan M. Pulst and has since been one of the most innovative journals in genome sciences. Over the past twenty years, genome science has been revolutionized by high-throughput sequencing and related omics technologies, alongside the development of bioinformatic and computational tools to analyze the large amounts of data they produce.

   Current Genomics has followed these technological advances very closely by publishing innovative articles in all areas of genome science and related fields, including research papers reporting on new and original data generated at the genome scale level, authoritative and comprehensive full-length or mini-review articles covering the latest developments, as well as position and opinion papers from internationally recognized experts addressing contemporary questions and issues.

   All things considered, we are grateful to the authors, readers, reviewers, and editorial staff members who have contributed to the success of the journal and look forward to continue providing essential readings in all areas of genome science.

